# Extrahepatic Replication and Genomic Signatures of the Hepatitis E Virus in the Kidney

**DOI:** 10.1111/liv.70183

**Published:** 2025-06-27

**Authors:** Avista Wahid, Nele Meyer, Christine Wundes, Lucas Hüffner, Saskia Janshoff, Nicola Frericks, Martina Friesland, Katja Dinkelborg, Elmira Aliabadi, Fenja Laue, Markus Cornberg, Benjamin Maasoumy, Birgit Bremer, Sven Pischke, Tobias Müller, Julian Zur Schulze Wiesch, Julia Benckert, Rainer G. Ulrich, Svenja Hardtke, Petra Dörge, Florian Vondran, Ansgar Lohse, Michael Peter Manns, Daniel Todt, Heiner Wedemeyer, Thomas Pietschmann, Eike Steinmann, André Gömer, Patrick Behrendt

**Affiliations:** ^1^ Institute for Experimental Virology TWINCORE, Centre for Experimental and Clinical Infection Research, a Joint Venture Between the Helmholtz Centre for Infection Research and the Hannover Medical School Hannover Germany; ^2^ Department of Molecular and Medical Virology Ruhr University Bochum Germany; ^3^ Herz‐ Und Diabeteszentrum Nordrhein‐ Westfalen Bad Oeynhausen Germany; ^4^ Department for Translational and Computational Infection Research Ruhr University Bochum Bochum Germany; ^5^ Department of Gastroenterology, Hepatology, Infectious Diseases and Endocrinology Hannover Medical School Hannover Germany; ^6^ Center for Individualised Infection Medicine (CiiM) Hannover Germany; ^7^ German Center for Infection Research (DZIF), HepNet Study‐House/German Liver Foundation Hannover Germany; ^8^ DZIF, Partner Site Hannover‐Braunschweig Germany; ^9^ University Medical Centre Hamburg‐Eppendorf Hamburg Germany; ^10^ DZIF, Partner Site Hamburg‐Lübeck‐Borstel‐Riems Germany; ^11^ Department of Gastroenterology and Hepatology Charité Campus Virchow‐Klinikum (CVK) Berlin Germany; ^12^ Institute of Novel and Emerging Infectious Diseases Friedrich‐Loeffler‐Institut, Federal Research Institute for Animal Health Greifswald Germany; ^13^ Regenerative Medicine and Experimental Surgery (ReMediES) Department of General, Visceral and Transplantation Surgery, Hannover Medical School Germany; ^14^ European Virus Bioinformatics Center Jena Germany

**Keywords:** compartmentalization, hepatitis E, kidney impairment

## Abstract

**Introduction:**

The hepatitis E virus (HEV; species *Paslahepevirus balayani*) is a common human pathogenic and zoonotic virus that can cause both acute fulminant and chronic hepatitis. Despite its reputation as a hepatotropic virus, HEV infection is also associated with a number of extrahepatic diseases, including kidney disorders. However, the extent to which HEV replicates in kidney cells remains unclear. The present study aims to investigate the capacity of HEV to propagate in kidney cells in vitro and to assess whether HEV displays mutational signatures that correlate with compartmentalisation in vivo.

**Methods:**

We use HEV cell culture models to study the replication cycle and the effect of antivirals in human kidney cell lines and primary cells. In addition, we identified patients with chronic HEV infection (*n* = 9) from which we then sequenced the viral RNA of urine, stools and plasma to analyse the viral sequence composition, to assess intra‐host diversity and compartmentalisation (*n* = 2).

**Results:**

A wide range of human kidney cell lines as well as primary cells supports viral entry, replication and propagation of HEV in vitro. Interestingly, the broad‐spectrum antiviral ribavirin was less effective in inhibiting HEV replication in some kidney cells. Sequencing of HEV RNA‐directed RNA polymerase coding region from plasma, stool and urine and subsequent phylogenetic analysis revealed diversification of HEV into tissue‐specific viral subpopulations. In particular, the viruses derived from urine were found to be distinct from those derived from plasma and stool.

**Conclusions:**

In conclusion, kidney cells support the propagation of HEV in vitro and exhibit reduced sensitivity to antiviral treatment. Furthermore, HEV patient‐derived sequences demonstrated compartmentalisation into distinct clusters that correlated with sample source. Collectively, these data indicate the potential for extrahepatic replication of HEV, which may result in clinically significant disease or serve as a reservoir for patient relapse.

**Trial Registration:** HepNet‐SofE study (NCT03282474)

**Summary**

- Hepatitis E virus (HEV) is known as a liver‐tropic virus but has also been associated with extrahepatic disease. Our study shows that HEV can also propagate in human kidney cells, where it responds differently to antiviral treatment. In addition, in two patients, the virus in the urine is genetically different from the virus in the blood and faeces. These findings suggest that HEV can replicate outside the liver, which could have implications for disease outcomes and treatment strategies.

## 1 Introduction

Hepatitis E virus (HEV, species *Paslahepevirus balayani*, family *Hepeviridae*) is a single‐stranded, hepatotropic RNA virus and is the most common cause of acute viral hepatitis worldwide, annually infecting more than 20 million people [1]. Although hepatitis E virus infection is typically self‐limiting and asymptomatic in healthy individuals, it can also cause persistent infection. Overall, HEV causes approximately 3.3 million symptomatic cases annually and about 70 000 fatalities [2]. Patients at risk of developing chronic infections include individuals that are immunocompromised, such as solid organ transplant recipients, stem cell transplant recipients or human immunodeficiency virus (HIV)‐infected individuals. Persistent infection is often associated with hepatitis, which promotes liver fibrosis and progression to end‐stage liver disease with the development of concomitant complications. In addition, HEV can cause acute liver failure in patients with underlying liver diseases.

Unfortunately, the treatment options for patients with symptomatic HEV infection are limited. For immunosuppressed transplant patients, reduction of immunosuppression is recommended, but this bears the risk of graft rejection. Off‐label use of the broad‐spectrum antiviral ribavirin (RBV) is recommended for these patients by the European Association for the Study of the Liver [3]. However, the efficacy of RBV is limited, showing ~80% sustained virologic response, possibly due to the emergence of resistance‐associated variants in patients [4, 5, 6]. In addition, many patients are ineligible for treatment due to kidney insufficiency, highlighting the urgent need for effective antivirals and vaccines to treat and prevent HEV infection.

Although HEV is known as a major cause of viral hepatitis, it has also been associated with extrahepatic manifestations, including neurological, pancreatitis, myositis and kidney disease [7]. However, a causal relationship between extrahepatic disease and viral infection has not been established yet. Possible mechanisms underlying these morbidities include indirect immune‐mediated damage, as suggested by Weber et al., where HEV capsid protein accumulates in glomeruli [8]. Alternatively, these morbidities may result from HEV replication in extrahepatic sites or other immune‐mediated processes [9]. For neuronal cells, it has been shown that HEV can infect and replicate in these cells in vitro: This provides a potential link to how neurological diseases may occur in patients and a model system to study these [10, 11, 12]. Many patients with HEV infection and kidney dysfunction have been identified [13, 14, 15, 16, 17, 18, 19, 20, 21, 22, 23, 24]. Clinically, these patients often present with glomerulonephritis, proteinuria, relapses of immunoglobulin A (IgA) nephropathy and cryoglobulinemia. Notably, HEV antigen and RNA have been detected in the urine of the patients [24, 25]. In addition, HEV antigen was also detected in the urine of patients who had already cleared chronic HEV infection from their plasma for several months [17]. Whether this is only non‐infectious antigen or representative of infectious virus particles from an extrahepatic site of replication remains elusive.

The current study investigated the replication capability of HEV in kidney cell lines and primary kidney cells in vitro. Furthermore, the efficacy of replication and the efficacy of RBV in different kidney cell lines was analysed and compared to the effect in hepatoma cells. Additionally, evolutionary signatures that could be linked to compartmentalisation in vivo were characterised.

## 2 Materials and Methods

### 2.1 Patient Samples

Plasma, stool and urine samples were collected from nine patients with chronic HEV‐3 infection who participated in the HepNet‐SofE study (NCT03282474) [26]. These samples were collected from patients who previously failed or were ineligible for antiviral therapy with RBV and at a time when the patients were not being treated with antiviral drugs for at least 3 months. Subsequently, the samples were employed for the assessment of clinical parameters and viral high‐throughput sequencing. Patient characteristics are listed in Table 1.

**TABLE 1:** Patients from the HepNet‐SofE Trial with chronic HEV infection. HEV viral load was measured from plasma, stool and urine. Creatine clearance rates were measured, and HEV subgenotype was determined from sequencing data.

Patient ID	HEV RNA plasma [IU/mL]	Stool RNA [IU/mL]	Urine RNA [IU/mL]	CreaClr [mL/min]	Genotype
P1	55 000	34 800 000	n.d.	41	HEV3c
P2	140 000	37 200 000	1 370 000	59	HEV3c
P3	970 000	14 500 000	n.d.	75.65	HEV3c
P4	6 000 000	46 500 000	1730	61.35	HEV3
P5	15 000 000	460 000 000	n.d.	64.93	HEV3c
P6	200 000	175 000 000	n.d.	52.94	HEV3l
P7	327 000	1 320 000	51 700	63.5	HEV3c
P8	29 821 000	12 100 000	n.d.	49.4	HEV3c
P9	1 713 000	210 000 000	22 600	70	HEV3c

### 2.2 Eukaryotic Cell Culture

Human kidney carcinoma cells (A498, BFTC‐909, 786–0), non‐cancer cells (HK‐2, 293 T) and primary human kidney cells mixed with primary human epithelial cells (ATCC‐PCS‐400‐012) were utilised for analysis. In addition, we used human hepatoma cell lines HepG2 and HepG2/C3A as controls. Media and supplements were applied for each cell line as recommended and indicated in Table S1. The cells were kept at 37°C and 5% CO_2_.

### 2.3 Hepatitis E Virus Production

Infectious HEV cell culture‐derived (HEVcc) particles were produced according to Todt et al. 2019 [27].

Briefly, target cells, either hepatoma or kidney cells, were electroporated with in vitro transcribed full‐length Kernow‐C1/p6 RNA or full‐length 83–2‐27 RNA. To obtain extracellular enveloped HEV (eHEVcc), the supernatant was collected at least 7 days post‐transfection and stored at 4°C until use. Intracellular non‐enveloped HEV (neHEVcc) was harvested from the cell lysate. This was achieved by trypsinisation of the cells, neutralisation in cell specific medium, centrifugation at 100 × g for 5 min and resuspension in cell‐specific medium. After three freeze–thaw cycles in liquid nitrogen, the cells were centrifuged at 10000 × g to separate the viral particles from the cell debris. The clarified supernatant was aliquoted and stored at −80°C.

### 2.4 Gaussia *Luciferase Reporter Assay*

To test the susceptibility of kidney cells to support HEV replication, in vitro‐transcribed RNA based on an HEV‐3 Kernow‐C1/p6, an HEV‐3 83–2‐27 or an HEV‐1 Sar55 replicon, in which parts of open‐reading frame 2 (ORF2) were replaced by Gaussia luciferase, was introduced into kidney or hepatoma cells by electroporation according to Todt et al. [28]. In addition, a replication‐deficient mutant in which the catalytically active site of the HEV‐3 Kernow‐C1/p6 replicon was mutated (GAA) was used as a control. Briefly, 2.5 × 10^6^ cells were resuspended in 380 μL Cytomix and electroporated with 2.5 μg in vitro‐transcribed HEV replicon RNA. Cells were electroporated using the Gene Pulser Xcell apparatus (Bio‐Rad) and seeded at a density of 40 000 cells/well, except of A498 which were seeded at 80,000 cells/well in a 96‐well format. RBV was used as a replication control and Puromycin as an additional background control. Both were added 4 h after electroporation. Supernatants were collected at 4, 24, 48 and 72 h after electroporation. *Gaussia* luciferase activity was determined by detection of luminescence using a Centro XS3 LB 960 luminometer (Berthold Technologies). The luciferase reaction was performed on a 96‐well LUMITRAC 600 plate by adding 60 μL coelenterazine substrate to 10 μL harvested cell culture supernatant per well, shaking for 1 s and reading for 1 s.

### 2.5 Infection Assay

To determine whether cells are susceptible to HEV infection, cells were seeded on a 24‐ or 96‐well plate at a cell‐specific density (Table S2). After 24 h, the medium was aspirated, and the cells were infected with intracellular HEV‐3 p6 virus at a multiplicity of infection (MOI) of 0.2 to 3.0 and with intracellular HEV‐3 83–2‐27 virus at a MOI of 0.1 to 1.0. Cells were incubated for 5 days before preparation for immunofluorescence staining and microscopic analysis. A human anti‐HEV antibody [29] was used to pre‐incubate HEVcc as a specificity control. The ORF2 protein was stained using a polyclonal HEV‐3 capsid‐specific rabbit serum (1:5000 in 5% horse serum), and a rabbit anti‐HEV antibody (concentration 0.1 μg/mL in 5% horse serum) was used [29, 30]. Of note, PHKC were supplemented with baricitinib, a JAK–stat inhibitor that modulates innate immune responses prior to inoculation.

### 2.6 Antigen Assay

To assess the ability of kidney and hepatoma cells to produce HEV antigen, cells were electroporated with full‐length HEV Kernow C1/p6 transcripts. After electroporation, the production of viral antigen was measured 24, 48 and 72 h later using enzyme‐linked immunosorbent assay (ELISA), which detects the HEV capsid protein [Wantai Biological Pharmacy Enterprise, Beijing, China, Catalogue #WE‐7596]. For this, both cell lysates and supernatants were harvested from electroporated cells.

### 2.7 Density Gradient Analysis

To analyse the biophysical properties of HEVcc produced in kidney cells, density gradient centrifugation was performed as follows: A stepwise iodixanol gradient was generated by diluting Optiprep (Sigma Aldrich) in diluent (= 0.85% NaCl +30 mM Tricine‐NaOH pH 7.4) to a final concentration of 40% (w/v). Fractions were then generated by mixing with CSM (= 0.85% NaCl +10 mM Tricine‐NaOH pH 7.4). After stacking the gradient fractions, 1 mL of virus stock was mixed with 2 mL of OptiPrep. The tubes were then centrifuged in an ultracentrifuge [Thermo] at 30000 rpm for 24 h. After centrifugation, 1 mL fractions of the gradient were collected and prepared for RNA extraction [Promega Maxwell 16 Viral Total Nucleic Acid Purification Kit, Promega Maxwell] and RT‐PCR [Roche Light Cycler 480I]. The refractive index of each fraction was measured using a refractometer to calculate densities.

### 2.8 Amplicon Generation and Sequencing

Total RNA was extracted from plasma, stool suspension or urine using the Cobas AmpliPrep Total Nucleic Acid Isolation Kit (Roche, Basel, Switzerland). Complementary DNA (cDNA) synthesis and amplicon generation were conducted in accordance with Todt et al. [6]. Briefly, cDNA was synthesised from 2 to 8 μL of purified total RNA using the SuperScript III first‐strand synthesis system (Life Technologies, Carlsbad, CA, USA). Amplicons were generated in a two‐step touchdown PCR using TaKaRa Ex Taq Hot Start Version polymerase (Dalian, China). The NEBNext Ultra II FS DNA Library Preparation Kit (NEB) was used to prepare sequencing libraries according to the manufacturer's recommendations. Library quality was determined with the Agilent High Sensitivity DNA Kit (Agilent Technologies, Waldbronn, Germany) and a 2100 Bioanalyzer Instrument (Agilent Technologies). All samples were normalised and quantified using the KAPA Library Quantification Kit for Illumina (Kapa Biosystems, Wilmington, MA, USA). Sequencing was conducted on a Illumina MiSeq using a 2 × 300 base pair (bp) paired‐end sequencing protocol.

### 2.9 Data Analysis

Data analysis was based on the pipeline described by Gömer et al. [31]. Briefly, raw reads were trimmed using Trimmomatic 0.39 and quality‐checked using FastQC (https://www.bioinformatics.babraham.ac.uk/projects/fastqc/). Subsequently, a consensus reference sequence was generated from plasma samples by first mapping sample reads to a HEV‐3c reference sequence (accession: FJ705359) and subsequently generating the consensus sequence using Sam2Consensus v2.0 (https://github.com/vbsreenu/Sam2Consensus). These sequences were subsequently utilised in downstream analysis as a reference for mapping via Tanoti and variant calling using Diversitools (https://github.com/josephhughes/DiversiTools) and vNvS tools (https://github.com/rjorton/vnvs). Amino acid substitutions were visualised on an alpha fold 2‐based model, which employed the HEV‐3 Kernow/p6 genomic content. The graphical visualisation of the model was conducted in ChimeraX v1.7. Haplotypes were generated using CliqueSNV (https://github.com/vtsyvina/CliqueSNV), which were used for phylogenetic and genome linkage analysis. Phylogenetic trees were computed with IQ‐tree2 [32] using the option best modelfind and 1000 bootstraps. To identify iSNVs for each haplotype, the patient's plasma consensus sequence was used as a reference sequence. Phylogenetic trees were visualised with in‐house *R* scripts, utilising the following packages: tidyverse, tibble, treeio, ggtree, phytools and cowplot.

### 2.10 Graphics and Statistics

Statistical analysis was conducted in *R* (version 4.3.1). Graphical visualisation was performed in *R* using the ggplot2 library or Adobe Illustrator.

## 3 Results

### 3.1 Kidney Cell Lines Support Complete HEV Replication Cycle In Vitro

**FIGURE 1:**
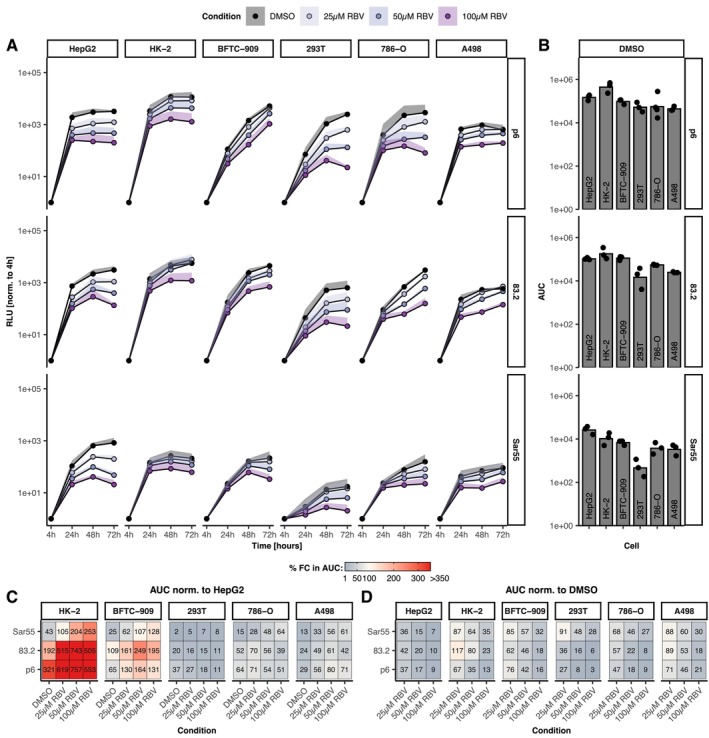
Recapitulation of the complete HEV replication cycle in human cells. (A) HEV replication in human renal cell lines using the Kernow‐C1/p6, 83–2‐27 and Sar55 replicon systems. Relative light units (RLU) were normalised to 4 h post‐electroporation (EPO) to quantify HEV replication. Ribavirin (RBV) was applied at concentrations of 25, 50 or 100 μM (purple shades) 4 h after electroporation. Dimethylsulfoxide (DMSO) was added at the same concentrations as a loading control (black). The lines represent data from at least three independent experiments, with the standard deviation shown as ribbon. (B) Area under the curve (AUC) quantification of replicative capacity over 72 h for DMSO control. (C) AUC normalisation of the replication signal to HepG2, allowing comparison of replication capacity between cell lines. (D) AUC normalisation to the DMSO loading control to assess cell‐type‐specific ribavirin inhibition.

**FIGURE 2:**
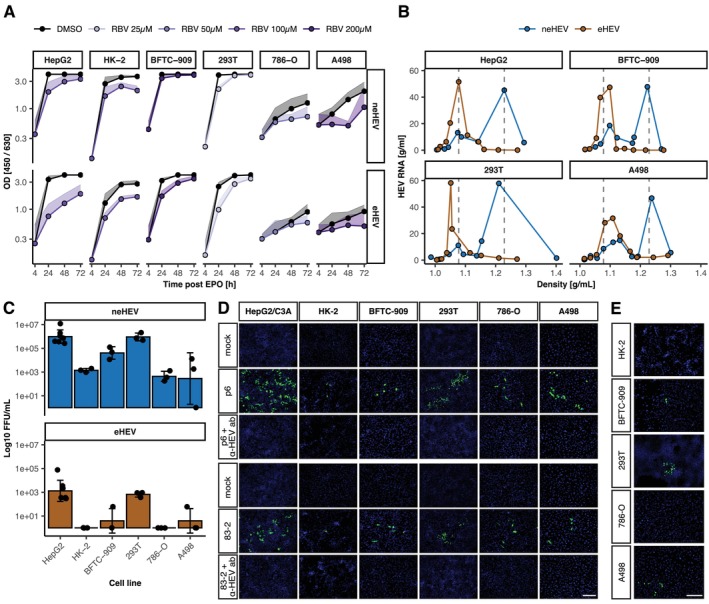
HEV can complete its full viral replication cycle in kidney cells. (A) Viral antigen production was measured after electroporation of viral RNA into kidney and liver cells using the Wantai antigen enzyme‐linked immunosorbent assay (ELISA). (B) Biophysical properties of the virus produced in different renal cell lines were assessed by measuring the RNA content after density centrifugation of cell lysates and supernatants. (C) Infectivity of virus particles produced in renal cell lines was assessed in HepG2/C3A cells by determining focus‐forming units (FFU) per mL of virus stock. (D) Susceptibility of renal cells to HEV infection was tested using neHEV particles produced in HepG2 cells. Five days after infection, the cells were immunostained for the ORF2‐encoded capsid protein (CP) using an anti‐capsid protein antibody. An anti‐HEV antibody (ab) at a concentration of 10 μg/mL was used as a specificity control. (E) Ability of neHEV produced in kidney cells to infect the same cell type was assessed.

To establish a potential causal relationship between clinical kidney symptoms and HEV infection in kidney cells, we sought to test whether HEV could recapitulate its complete replication cycle in kidney cell lines in vitro. To investigate HEV replication in kidney cells, we tested five human kidney cell lines and compared them to HepG2 hepatoma cells by electroporating Kernow‐C1/p6, 83–2‐27 and Sar55 replicon RNA (Figure 1A,B). All kidney cell lines showed a strong, time‐dependent increase in luciferase signal above background levels (4 h) for all isolates tested. Notably, HEV replication levels in certain kidney cell lines, particularly HK‐2 and BFTC‐909, were comparable to or up to 3.2‐fold higher than those in HepG2 cells for p6 and 83–2‐27, whereas Sar55 did not show this increase (Figure 1A–C). Replication kinetics also revealed differences in RBV efficacy between the kidney cell lines. When normalised to their respective DMSO controls, RBV inhibition of HEV replication was weakest in BFTC‐909 and HK‐2 cells. At 50 μM, HEV replication was reduced fourfold and twofold, respectively, at 72 h post‐electroporation, compared to a ninefold reduction in HepG2 cells, without significant loss of cell viability (Figure 1D, FiguresS1–S3). We then investigated the antigen production by these cells: For this, the in vitro‐transcribed authentic Kernow‐C1/p6 RNA was electroporated into HepG2 cells and the kidney cells (Figure 2A). Antigen production from cell lysate (intracellular, non‐enveloped virus, neHEV) and supernatant (extracellular, enveloped virus, eHEV) was measured at 4, 24, 48 and 72 h post electroporation using a Wantai antigen ELISA and normalised to levels at 4 h. All cell lines produced measurable virus antigen in the ELISA which was inhibited by RBV. Noteworthily, reduction by RBV was in some cell lines, including BFTC‐909, only minor. Antigen production was especially high in HK‐2, BFTC‐909 and 293 T cells which showed a similar level of antigen production as HepG2 cells. In contrast, 786‐O and A498 cells produced less antigen. We then tested whether the viral particles produced recapitulated biophysical properties of those produced in hepatoma cells by performing density gradient centrifugation (Figure 2B). In order to achieve this objective, two cell lines were selected that exhibited high levels of replication and a high capacity to produce antigens (BFTC‐909 and 293 T). One cell line was also included, as it demonstrated a lower level of replication and antigen production efficiency (A498). It was observed that, similar to the virus produced in HepG2 cells, virus produced in BFTC‐909 and A498 cells exhibited peaks at a density of 1.08 g/mL, representing extracellular virus, and a peak at 1.23 g/mL, representing intracellular virus. In contrast, the virus particles produced in 293 T cells exhibited a slight shift towards lower densities. As a next step, we inoculated HepG2 cells with the virus produced in the kidney cells to assess their infectivity (Figure 2C). All kidney cell lines supported production of infectious HEV as evidenced by capsid protein (CP)‐positive target cells (HepG2/C3A), with titres ranging from approximately 6 × 10^2^ to 1.1 × 10^6^ FFU/mL for neHEV. Here, 293 T cells have produced similar amounts of infectious virus to HepG2 cells (2.4 × 10^6^ FFU/mL), while virus produced in HK‐2 cells had a lower titre (1.5 × 10^3^ FFU/mL). We then tested the ability of p6 and 83‐2‐27 to infect kidney cells using virus produced in HepG2 cells (Figure 2D). All cell lines supported infection with neHEV by both isolates, while infection was effectively inhibited by a neutralising antibody in the negative control. To confirm that HEV can complete its full replication cycle in kidney cells, we generated virus in kidney cells and subsequently inoculated both liver and kidney cell lines. Following electroporation, HepG2/C3A cells were inoculated to verify successful virus production, as indicated by positive ORF2 staining in all kidney‐derived virus samples (Figure S4). Simultaneously, we inoculated the same kidney cell lines used for virus production (Figure 2E). With the exception of HK‐2 and 786‐O cells, all kidney cell lines supported HEV infection, as demonstrated by positive immunofluorescence (IF) staining for the ORF2‐encoded capsid protein (CP). As before, the addition of neutralising antibodies significantly reduced infectivity. Interestingly, HK‐2 cells, showed less infected cells overall despite supporting HEV replication and antigen production at high levels. To demonstrate that viral entry and CP production are not exclusive to kidney cell lines, we employed more authentic primary human kidney cells (PHKC) for neHEV infection. Indeed, PHKCs were also susceptible to HEV infection after inoculation with HEVcc, but only when baricitinib was used to blund innate immune responses (Figure S5). In conclusion, we have shown that HEV can recapitulate its entire viral replication cycle in several kidney cell lines in vitro, including viral entry, replication and assembly and release of infectious virus. Furthermore, we have demonstrated that primary kidney cells are also susceptible to HEV infection.

### 3.2 Patients With Chronic HEV Show Viral RNA Load in Plasma, Stool and Urine

To gain a better understanding of the HEV infection dynamics in patients with chronic infection we examined the samples obtained from patients who took part in the SofE trail [26]. Due to the prolonged infection that these patients had suffered, with the possible acquisition of viral mutations leading to adoption to the host, they might also have acquired extrahepatic infection. Samples were collected from these patients after they had been off ribavirin treatment for at least 3 months, yet remained viremic. Subsequently, we then tested these patients for viral RNA load in plasma, stool and urine (Table 1). Four patients P2, P4, P7 and P9 tested positive for HEV RNA in all three samples. For two of these patients, P2 and P7, urine, plasma and faecal samples were available for analysing viral compartmentalisation, as presented in the next paragraph. Patient P2 was kidney‐transplanted and received immunosuppressant drugs (Tacrolimus, Mycophenolate and Prednisolon). Patient P7 was a multivisceral transplant patient, who was also immunosuppressed (Sirolimus and Tacrolimus). Both patients were HEV‐positive for over a year and received prior treatment with RBV for prolonged time (~1.3 years, 3.3 years) which did not lead to viral clearance.

### 3.3 High‐Throughput Sequencing of Plasma, Stool and Urine Samples From Patients Reveals HEV Subpopulation Compartmentalisation Indicative of Extra‐Hepatic Replication Sites

**FIGURE 3:**
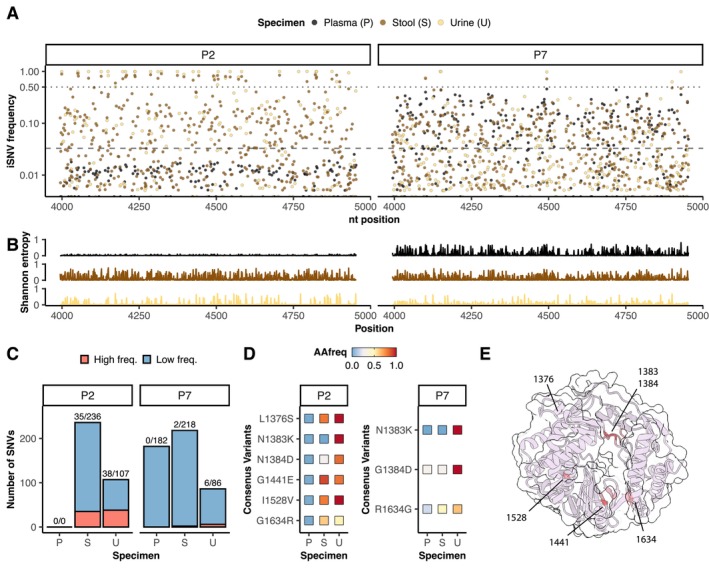
Virus RNA sequencing shows divergence of HEV populations in samples from different sources. (A) Intra‐host single nucleotide frequencies (iSNV) of the RNA‐directed RNA polymerase (RdRp) coding region of virus populations sampled from plasma (*P*, black), stool (*S*, brown) and urine (*U*, yellow) of patient 2 (P2) and patient 7 (P7). (B) Nucleotide sequence divergence summarised as Shannon entropy. (C) Quantification of low frequency (above 3.28%, blue) and high frequency (above 50%, red). (D) Frequency of amino acid (AA) substitutions within the RdRp of HEV strains in the three samples of both patients. (E) Representation of amino acid substitutions on an alpha‐fold‐2‐generated 3D structure model of the viral polymerase.

**FIGURE 4:**
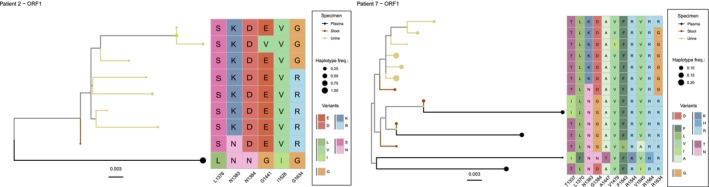
Phylogenetic reconstruction from haplotype data. NGS reads were used to reconstruct haplotypes using CliqueSNV. A multiple sequence alignment was constructed using Clutal Omega. Phylogenetic trees were reconstructed using the maximum likelihood method implemented in IQ‐tree 2. The trees were then visualised in *R* using the ggtree package. Supported bootstrap values (≥ 80%) are visualised with green dots on the parent branches. Finally, heatmaps were created to visualise amino acid substitutions in comparison to the reference sequence. The colour coding of the heatmap depicts chemical properties, with negative charges (D, E), positive charges (R, H, K), polar‐uncharged (S, T, N), non‐polar‐uncharged (A, F, I, L, V) and others (C, P, G) represented by different colours. The colour of the dots indicates the specimen origin, with yellow dots representing urine, brown dots representing stool and black dots representing plasma. The size of the dots indicates the frequency of haplotypes.

We then hypothesised that if HEV replicates in different compartments, it may independently accumulate mutational signatures in each compartment during chronic infection, either through neutral or selective evolution. To test this hypothesis, we performed high‐throughput sequencing of viral subpopulations from plasma, stool and urine (Figure 3). Viral populations identified in plasma should represent viruses that originated in the liver and were secreted basolaterally from liver cells; viruses found in stool should recapitulate viral replication in the gut as well as in the liver, secreted through the bile duct; and viruses identified in urine should originate in the kidney. Viral sequencing was conducted on the viral RNA‐dependent RNA polymerase (RdRp) domain with an overall coverage of 3714 (standard deviation 656, Figure S6). To identify sequence variations between the HEV subpopulations in the three compartments we used the consensus sequence of plasma originating virus as reference for the analysis. By this way, we were able to spot differences in viral genome composition in regard to the plasma sample. Overall, viral subpopulations were divergent with multiple intra‐host single nucleotide variants (iSNVs) at low (below 50%) to high frequency (above 50%) in the two patients and the three compartments (Figure 3A). We additionally computed Shannon entropy as measure of diversity which was elevated throughout the RdRp without apparent peaks in diversity that may represent hotspots for diversity (Figure 3B). However, viral subpopulations in the plasma of patient P2 were much less divergent compared to those in patient P7. Additionally, we quantified the number of low frequency variants above the variant calling threshold of 3.287% and high frequency variants above 50% that represent changes in the consensus sequence (Figure 3C). This quantification showed that viral subpopulations diverged between the different compartments as between 86 to 236 low frequency iSNVs and 2 to 38 high frequency iSNVs were identified in the two patients. To get an insight into selection pressure that may lead to substitutions on the amino acid level we quantified changes in the frequency of amino acid variants (Figure 3D). Interestingly, we were able to identify six unique amino acid variants that were present at different frequency between the compartments in the two patients, including variants that are associated with RBV treatment failure: N1383K, N/G1384D and G1634R. As of yet, it remains unknown whether the other variants detected (L1376S, G1441E, I1528V) have an influence on antiviral resistance or adaptation to replication site specific conditions. Visualising these variants on an alpha fold generated RdRp 3D‐reconstitution demonstrated that the variation at amino acid position 1528 was concentrated in the vicinity of the nucleic acid entry site of the RdRp, while position 1376 was situated at the side of the RdRp model (Figure 3E). Subsequently, the aim was to ascertain which mutations may be present on the same genome. This was achieved by reconstructing viral genome haplotypes from HTS‐derived nucleotide sequences using CliqueSNV (Figure 4). These haplotypes were then used to infer phylogenetic trees for each patient using the maximum likelihood method, which were consistent with the data presented in Figure 3. They demonstrated that the RdRp‐encoding haplotypes from urine clustered together and were thus distinct from the viral haplotypes from stool or plasma. The tracing of amino acid substitutions demonstrated that lysine (K) at position 1383 appeared to be a unique feature of the urine‐derived virus in both patients, whereas asparagine (N) was the predominant amino acid in the stool‐ or plasma‐derived virus. In addition to the RdRp amplicon, we employed amplicon sequencing on the genomic region encoding the N‐terminal part of the capsid protein and ORF3 encoding protein (Figure S7). For patient P7, we were able to retrieve amplicon sequencing data for all three specimens. However, for patient P2, amplicon generation failed for serum, which is why for the analysis we used stool as a reference. As with the RdRp amplicon, genomic variability at the nucleotide level was detected for all specimens (Figure S7A,B). The number of low‐frequency iSNVs was similar in stool for both patients: 174 or 170 for P2 and P7, respectively. However, in P7, more high‐frequency iSNVs were detected, in comparison to P2. This trend was also observed in urine, where P2 exhibited a lower prevalence of high‐frequency iSNVs (7 out of 18) in comparison to P2 (30 out of 68). When analysing the number of amino acid substitutions in the capsid protein‐encoding region, a similar pattern was observed: P7 had a higher number of sites with highly variable residues (eight residues) in comparison to P2 (three residues) (Figure S7D). The substitutions observed in P2 were exclusively at the consensus frequency in urine, but not in stool. For patient P7, P68S and V267A were exclusively at the consensus level in urine. Of all variable residues within the capsid region, only P68S was present in both patients. As the region encoding the ORF3 and ORF2 genes overlap, we were also able to analyse amino acid substitutions within the ORF3 protein (Figure S7D). In total, six different sites exhibited substitutions, none of which were identical between the two patients. As observed in the capsid encoding region, the substitutions in P2 were at a high frequency only in urine and not in stool. For P7, only the Y71C substitution was at high frequency in urine and not in any of the other specimens, while P72R and L87S were observed in stool and urine. In conclusion, the sequencing data revealed genomic signatures that were consistent with their specimen, suggesting that HEV subpopulations diverge within the host depending on the viral replication site.

## 4 Discussion

The primary site of HEV replication is the liver – with high viral loads and accumulation of liver damage that can lead to fibrosis or end‐stage liver disease. Nevertheless, acute or chronic HEV infection are associated with relevant morbidity at extrahepatic sites that are not well understood. How these disorders arise and what influence they have on disease progression remains difficult to study in the absence of appropriate cell culture or animal models. Here, we show that HEV can fully satisfy the requirements for entry, replication and propagation in kidney cells, allowing the study of replication‐associated pathology and immune responses in vitro. Given the variety of extrahepatic diseases associated with HEV, such in vitro models may prove valuable in describing the pathology and identifying much‐needed effective antivirals.

In fact, HEV has been associated with a variety of extrahepatic disorders. Neurological manifestations include Guillain‐Barré syndrome (GBS) and neuralgic amyotrophy (NA) [33]. Similar to this study, HEV has been shown to replicate and infect neuronal cell lines in vitro [10, 11, 12]. It has also been shown that HEV can enter, replicate, and propagate in placenta‐derived JEG‐3 cells [34] and primary intestinal cells [35], highlighting the flexibility of HEV replication in different cell types. Although there are numerous other extrahepatic manifestations of HEV, it has not been demonstrated that the replication cycle of HEV can be completed in all of these cell types. Furthermore, it should be noted that these are only in vitro model systems and a causal relationship between clinical symptoms and direct HEV replication remains elusive. Larger patient cohorts and animal models will be needed to address these issues.

Both acute and chronic HEV infections have been associated with various renal dysfunctions, ranging from transient dysfunction to severe renal damage [7, 9, 14, 15, 36] with HEV clearance often associated with improved renal function [14, 18, 19]. However, it remains unclear why only a subset of patients with acute or chronic HEV infection develop extrahepatic disease or shed HEV RNA in their urine, a phenomenon previously observed in other studies [17]. Many questions remain about the link between HEV and extrahepatic replication, including how HEV infiltrates non‐hepatic organs and what predisposes certain patients to extrahepatic manifestations. One possibility is that extrahepatic damage may result from direct HEV replication in affected tissues or from infection‐induced immune‐mediated responses leading to tissue injury. HEV antigen has been readily detected in patients, not only during ongoing infection but also months after clearance of HEV infection from plasma [36]. It has therefore been proposed that non‐infectious, free ORF2‐encoded capsid protein is trapped in the glomeruli and causes kidney damage. Capsid protein has been shown to be highly stable, secreted in excess and to act as an immunogenic decoy [15, 37, 38, 39]. Viral RNA has also been detected in urine samples from patients, albeit at lower levels [9], and in animal experiments with cynomolgus monkeys [17]. These in vivo experiments highlighted the importance of HEV in urine, as HEV transmission from an infected to a naive monkey could be achieved by urinary inoculation, suggesting that infectious virus can be excreted in urine [17]. Furthermore, HEV antigen has been detected in kidney tissue sections and associated with pathological changes manifested as immune cell infiltration not only in experimentally infected monkeys [17] but also in rabbits [40, 41], gerbils [42] and pigs [43]. Comparable evidence was found in the histopathology of a patient with acute HEV infection showing crescentic and membranoproliferative glomerulonephritis [18].

Our clinical data from nine patients showed that HEV RNA can be detected in urine and that generating amplicons for next‐generation sequencing to analyse the composition of the virus population is possible. Of note, we were only able to sample and generate amplicons from two patients, likely due to low viral titres and high viral diversity in these samples. Therefore, future studies with larger cohorts of patients are needed to better understand the extent of HEV adaptation to extrahepatic sites, such as the kidney, and the factors that enable HEV infiltration, including the frequency of such events. Sequencing data from plasma, stool and urine samples from two patients showed that HEV sequences varied depending on the sample type. This finding supports the possibility of an extrahepatic replication site to which HEV may have adapted. Similar observations have been reported in previous studies where HEV compartmentalisation was detected in cerebrospinal fluid and post‐mortem brain tissue [44, 45, 46, 47] in patients with chronic HEV infection. In addition, evidence of HEV compartmentalisation in multiple tissues has been demonstrated in an in vivo porcine infection model [48]. Similar approaches have been used to show that hepatitis C virus (HCV) is compartmentalised in extrahepatic reservoirs [49, 50]. Their identification has proven to be critical in a clinical setting, as it has been shown that virus re‐emergence and treatment relapse can be facilitated by compartmentalised viral subpopulations [49]. Although short read sequencing provides an accurate and detailed overview of sequence polymorphisms in viral genomes present in clinical samples, it may also be prone to errors and uncertainties, e.g., the genome‐wide coupling of single‐nucleotide polymorphisms located at different genome positions. To overcome these limitations, future studies should confirm our findings in larger patient cohorts. The investigation of larger cohorts would also permit the examination of the prerequisites for HEV infiltration into the kidney and the frequency with which this occurs. Furthermore, the study could be extended to encompass host genetics, with a view to identifying host‐related factors. In addition, future studies should also examine HEV sequence polymorphisms at different sites in the genome and their coupling, with a potential to provide insights into tissue‐specific adaptation. The CP‐coding sequence could, for example, influence tissue‐specific infiltration or immune interaction. Nevertheless, the absence of full‐length sequencing methods that permit the sequencing of the entire genome from samples with low viral loads represents a significant challenge.

From a clinical perspective, the significance of extrahepatic replication is not only in terms of symptomatic alterations in patients but may also complicate treatment efficacy. Our in vitro data suggest that the antiviral efficacy of RBV is significantly reduced in kidney cell lines, which may be explained by two potential mechanisms: (i) Ribavirin requires phosphorylation by intracellular enzymes to convert the prodrug to its active form, a process that may vary between cell types due to differences in enzyme expression or activity [51, 52]. (ii) Intracellular activation of RBV may also depend on the efficiency of its uptake, as variability in nucleoside transporter expression or activity may influence prodrug influx and consequently its therapeutic efficacy [53]. Consequently, extrahepatic sites may serve as a reservoir for HEV that is difficult to treat with current antiviral agents and from which HEV may relapse after plasma clearance and treatment cessation. However, further investigations are needed to determine the exact mechanism and the extent to which it applies in vivo.

In conclusion, our findings demonstrate that HEV can complete its entire replication cycle in kidney cell lines in vitro and that in vivo HEV subpopulations exhibit mutational signatures indicative of compartmentalisation. These observations will be crucial in establishing a link between clinically relevant kidney diseases and HEV replication. Furthermore, our results indicate that RBV efficacy is substantially reduced in kidney cell lines, which may have significant clinical implications for antiviral treatment.

## Author Contributions

Conceptialisation and Design E.S., A.G., P.B.; Investigation and Visualisation: A.W., N.M., C.W., L.H., S.J., N.F., M.F., K.D., E.A., F.L., M.C., B.M., B.B., S.P., T.M., J.z.S.W., J.B., R.G.U., S.H., P.D., F.V., A.L., M.P.M., D.T., H.W., T.P., E.S., A.G., P.B.; Formal Analysis: A.W., N.M., A.G., P.B.; Drafting of the manuscript: A.W., N.M., E.S., A.G., P.B.; Supervision and funding acquisition: E.S., P.B.; Project administration: P.B.; Critical revision and editing of the manuscript: all authors.

## Ethics Statement

The authors have nothing to report.

## Consent

The authors have nothing to report.

## Conflicts of Interest

The authors declare no conflicts of interest.

## Supporting information


**Figure S1.** Replicative capacity of HEV. Replication capacity was measured by quantifying area under the curve (AUC) at 4, 24, 48 and 72 h. The dashed line represents the puromycin control. Each point corresponds to one biological replicate. The Kernow/C1 p6 GAA mutant was included as a negative control.
**Figure S2**. Cell viability of human kidney cell lines. The results of the potential cytopathic effect of ribavirin of different concentrations was measured in comparison to the DMSO control set to 1. Error bars indicate the standard deviation of three independent experiments. Dashed line set to 1.
**Figure S3**. Efficacy of ribavirin in kidney‐derived cell lines to suppress HEV replication. The efficacy of ribavirin was quantified as the difference of replication compared to the DMSO control. The fold change in replication between the DMSO control and cells treated with 25 μM, 50 μM or 100 μM RBV, at 72 h post electroporation, was calculated and is indicated in the boxes. Negative values indicate inhibition of replication.
**Figure S4**. Recapitulating the full HEV replicating cycle in kidney cells. Virus produced in kidney cell lines was used for infection of HepG2/C3A cells and the same cell lines with neHEV. Five days after infection, the cells were immunostained for the ORF2‐encoded capsid protein using an anti‐capsid protein antibody. As negative control, a neutralising anti‐HEV antibody at a concentration of 10 μg/mL was used. Scale bar denotes 200 μm.
**Figure S5**. Susceptibility of primary human and porcine kidney cells to HEV infection. Primary porcine cells (PHKC) were inoculated with HEV, and infectivity was determined by immunofluorescence staining of the capsid protein. Infection was done with non‐enveloped HEV or enveloped HEV. Specificity was controlled by applying 1:100 IgG positive serum. Inlets show infected cells.
**Figure S6**. Sequence coverage of the high‐throughput sequencing data for the three specimens. Coverage was determined for amplicons of patient 2 (P2) and patient 7 (P7) within the RNA‐directed RNA polymerase coding region on nucleotide (nt) sequences.
**Figure S7**. Virus RNA sequencing from ORF2 and ORF3 encoding regions. HEV ORF2 and ORF3 show mutational signatures of compartmentalisation from different sources. (A) Intra‐host single nucleotide frequencies (iSNV) of the N‐terminal part of the ORF2 and ORF3 coding region of virus populations sampled from plasma (*P*, black), stool (*S*, brown) and urine (*U*, yellow) of patient 2 (P2) and patient 7 (P7). (B) Nucleotide sequence divergence summarised as Shannon entropy. (C) Quantification of low frequency (above 3.28%, blue) and high frequency (above 50%, red). (D) Frequency of amino acid (AA) substitutions within the ORF2 region (two left panels) and ORF3 (two right panels) of HEV strains in the three samples of both patients.
**Table S1**. Cell media composition.
**Table S2**. Cell seeding densities.

## Data Availability

The data that support the findings of this study are openly available on the GEO https://www.ncbi.nlm.nih.gov/geo/query/acc.cgi?acc=GSE300759.
