# Supplementary material for: Extrahepatic Replication and Genomic Signatures of the Hepatitis E Virus in the Kidney
Source: Liver Int. 2025 Jun 27;45(7):e70183. doi: 10.1111/liv.70183 (PMC12203452; doi:10.1111/liv.70183)
Supplement: Supplementary file 1 — Figure S1. Replicative capacity of HEV. Replication capacity was measured by quantifying area under the curve (AUC) at 4, 24, 48 and 72 h. The dashed line represents the puromycin control. Each point corresponds to one biological replicate. The Kernow/C1 p6 GAA mutant was included as a negative control. Figure S2. Cell viability of human kidney cell lines. The results of the potential cytopathic effect of ribavirin of different concentrations was measured in comparison to the DMSO control set to 1. Error bars indicate the standard deviation of three independent experiments. Dashed line set to 1. Figure S3. Efficacy of ribavirin in kidney‐derived cell lines to suppress HEV replication. The efficacy of ribavirin was quantified as the difference of replication compared to the DMSO control. The fold change in replication between the DMSO control and cells treated with 25 μM, 50 μM or 100 μM RBV, at 72 h post electroporation, was calculated and is indicated in the boxes. Negative values indicate inhibition of replication. Figure S4. Recapitulating the full HEV replicating cycle in kidney cells. Virus produced in kidney cell lines was used for infection of HepG2/C3A cells and the same cell lines with neHEV. Five days after infection, the cells were immunostained for the ORF2‐encoded capsid protein using an anti‐capsid protein antibody. As negative control, a neutralising anti‐HEV antibody at a concentration of 10 μg/mL was used. Scale bar denotes 200 μm. Figure S5. Susceptibility of primary human and porcine kidney cells to HEV infection. Primary porcine cells (PHKC) were inoculated with HEV, and infectivity was determined by immunofluorescence staining of the capsid protein. Infection was done with non‐enveloped HEV or enveloped HEV. Specificity was controlled by applying 1:100 IgG positive serum. Inlets show infected cells. Figure S6. Sequence coverage of the high‐throughput sequencing data for the three specimens. Coverage was determined for amplicons of patient 2 (P2) [file LIV-45-0-s001.docx]

**Extrahepatic replication and genomic signatures of the hepatitis E virus in the kidney**

Avista Wahid^1,*^, Nele Meyer^1,*^, Christine Wundes^1^, Lucas Hüffner^1^, Saskia Janshoff^2,3,4^, Nicola Frericks^2^, Martina Friesland^1^, Katja Dinkelborg^1,5,6^, Elmira Aliabadi^1^, Fenja Laue^1^, Markus Cornberg^5,6,7,8^, Benjamin Maasoumy^5, 8^, Birgit Bremer^5^, Sven Pischke^9,10^, Tobias Müller^11^, Julian zur Schulze Wiesch^9,10^, Julia Benckert^11^, Rainer G. Ulrich^10,13^, Svenja Hardtke^7,9,10^, Petra Dörge^5,7^, Florian Vondran^8,12^, Ansgar Lohse^,9,10^, Michael Peter Manns^5,8^, Daniel Todt^2,4,14^ , Heiner Wedemeyer^5,8^, Thomas Pietschmann^1,8^, Eike Steinmann^2^, André Gömer^2,#^, Patrick Behrendt^1,4,7,#^

^1^ Institute for Experimental Experimental Virology, TWINCORE, Centre for Experimental and Clinical Infection Research, a joint venture between the Helmholtz Centre for Infection Research and the Hannover Medical School, Hannover, Germany.

^2^ Department of Molecular and Medical Virology, Ruhr University Bochum, Germany;

^3^ Herz- und Diabeteszentrum Nordrhein- Westfalen, Bad Oeynhausen, Germany

^4^ Department for Translational and Computational Infection Research, Ruhr University Bochum, Bochum, Germany

^5^ Department of Gastroenterology, Hepatology, Infectious Diseases and Endocrinology, Hannover Medical School, Hannover, Germany;

^6^ Center for Individualised Infection Medicine (CiiM), Hannover, Germany;

^7^ German Center for Infection Research (DZIF), HepNet Study-House/German Liver Foundation, Hannover, Germany,

^8^ DZIF, Partner Site Hannover-Braunschweig, Germany;

^9^ University Medical Centre Hamburg-Eppendorf, Hamburg, Germany;

^10^ DZIF, Partner Site Hamburg-Lübeck-Borstel-Riems, Germany;

^11^ Department of Gastroenterology and Hepatology; Charité Campus Virchow-Klinikum (CVK), Berlin, Germany,

^12^ Regenerative Medicine and Experimental Surgery (ReMediES), Department of General, Visceral and Transplantation Surgery, Hannover Medical School, Germany;

^13^ Institute of Novel and Emerging Infectious Diseases, Friedrich-Loeffler-Institut, Federal Research Institute for Animal Health, Greifswald-Insel Riems, Germany.

^14^ European Virus Bioinformatics Center, Jena, Germany

^*^ Shared first authorship

^#^ Shared last authorship

**Corresponding author:**

Name: Patrick Behrendt

Email: Behrendt.Patrick@mh-hannover.de

Name: André Gömer

Email: andre.goemer@rub.de


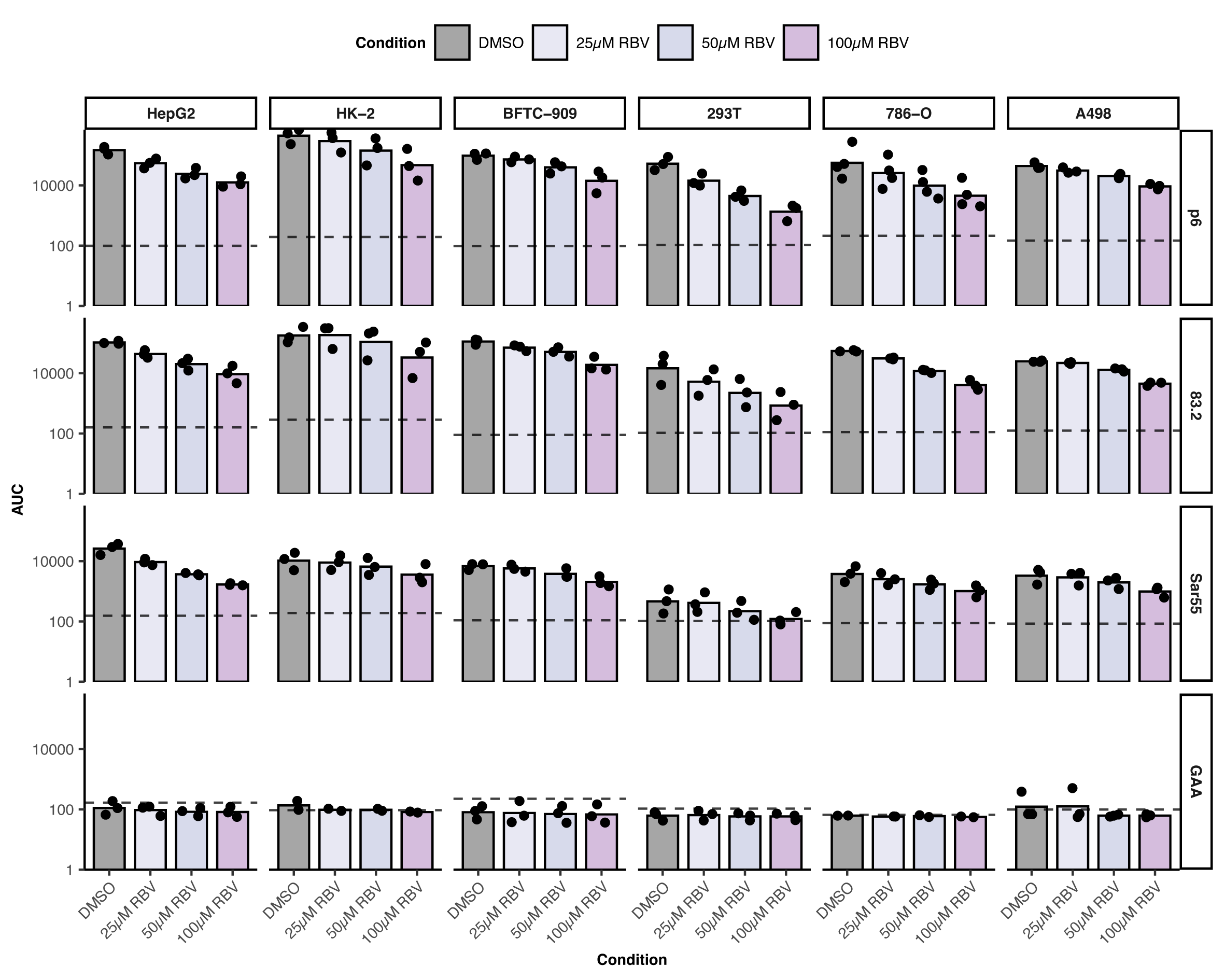


**Figure S1: Replicative capacity of HEV.** Replication capacity was measured by quantifying area under the curve (AUC) at 4, 24, 48 and 72 hours. The dashed line represents the puromycin control. Each point corresponds to one biological replicate. The Kernow/C1 p6 GAA mutant was included as a negative control.


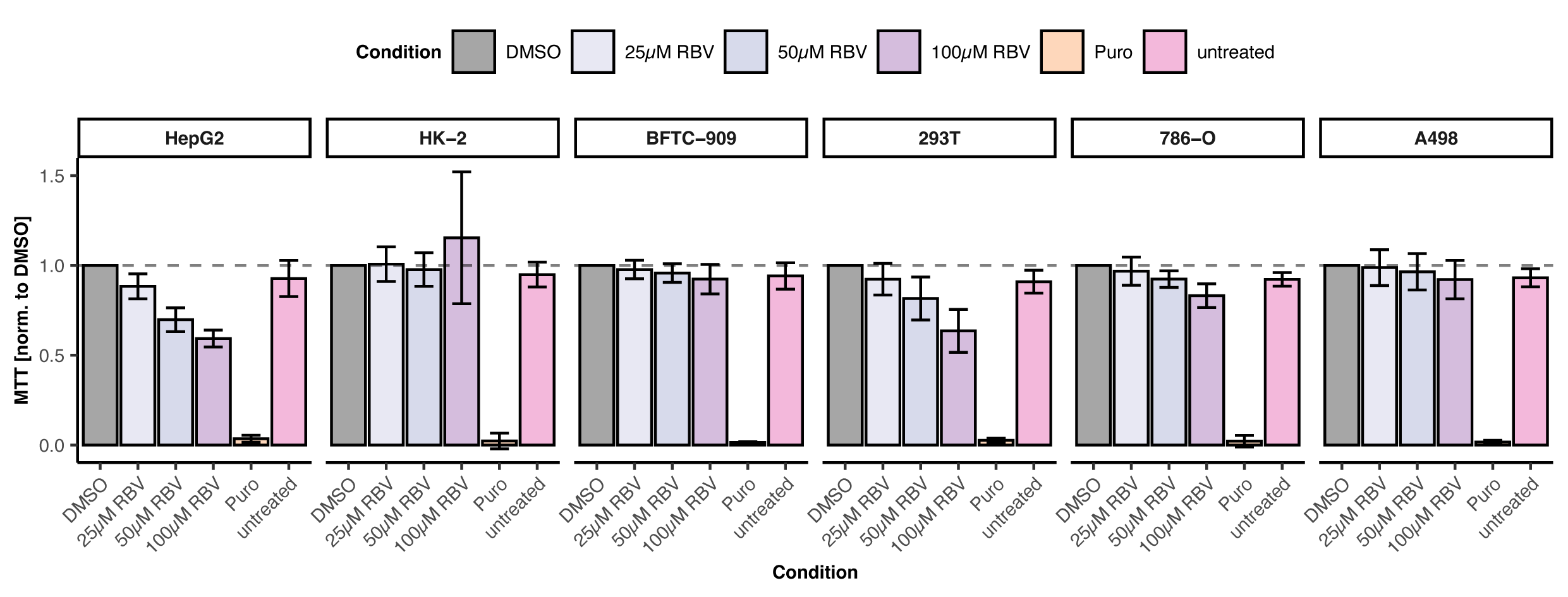


**Figure S2: Cell viability of human kidney cell lines.** The results of the potential cytopathic effect of ribavirin of different concentrations was measured in comparison to the DMSO control set to 1. Error bars indicate the standard deviation of three independent experiments. Dashed line set to 1.


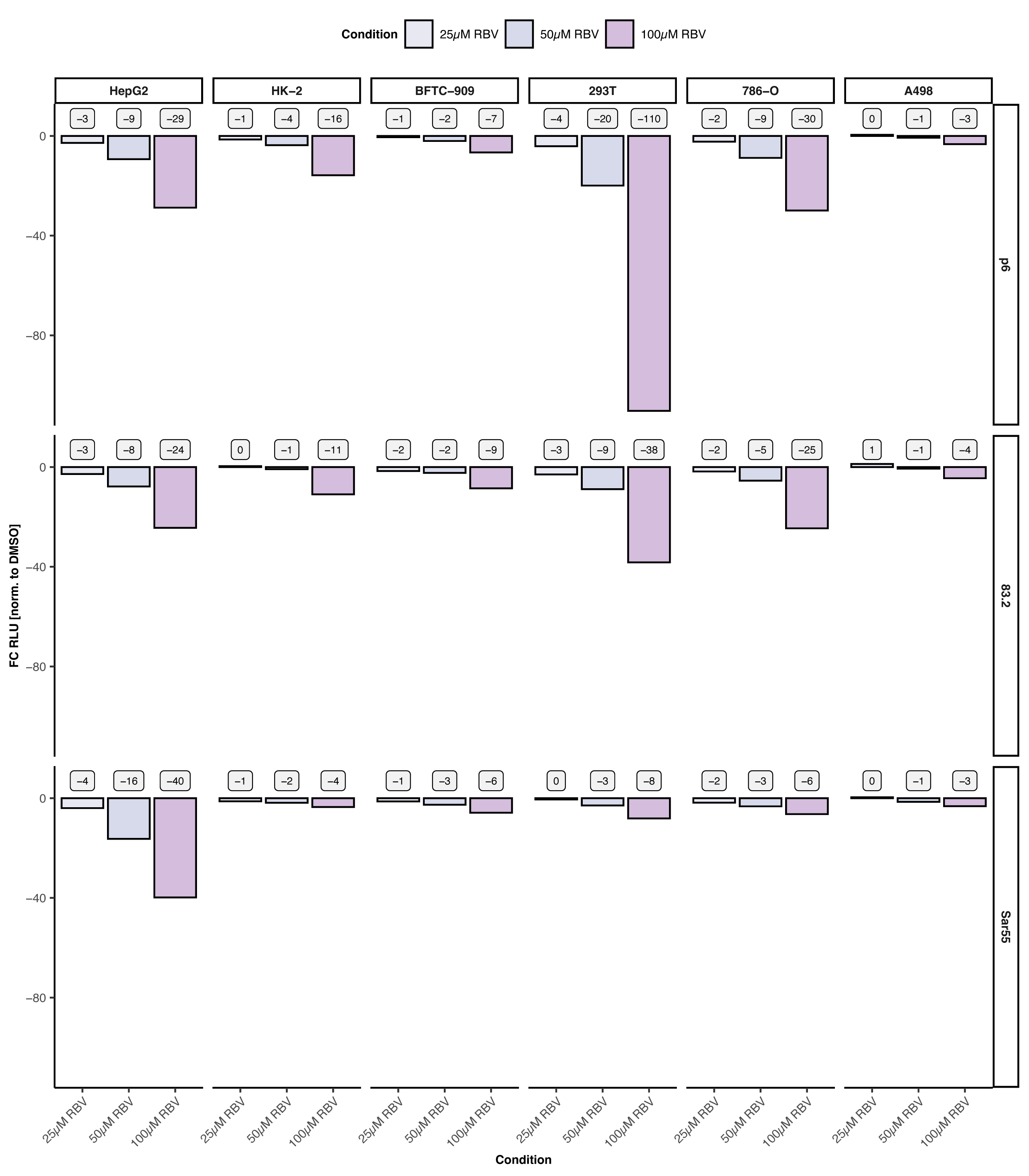


**Figure S3: The efficacy of ribavirin in kidney-derived cell lines to suppress HEV replication.** The efficacy of ribavirin was quantified as the difference of replication compared to the DMSO control. The fold change in replication between the DMSO control and cells treated with 25 µM, 50 µM or 100 µM RBV, at 72 hours post electroporation, was calculated and is indicated in the boxes. Negative values indicate inhibition of replication.


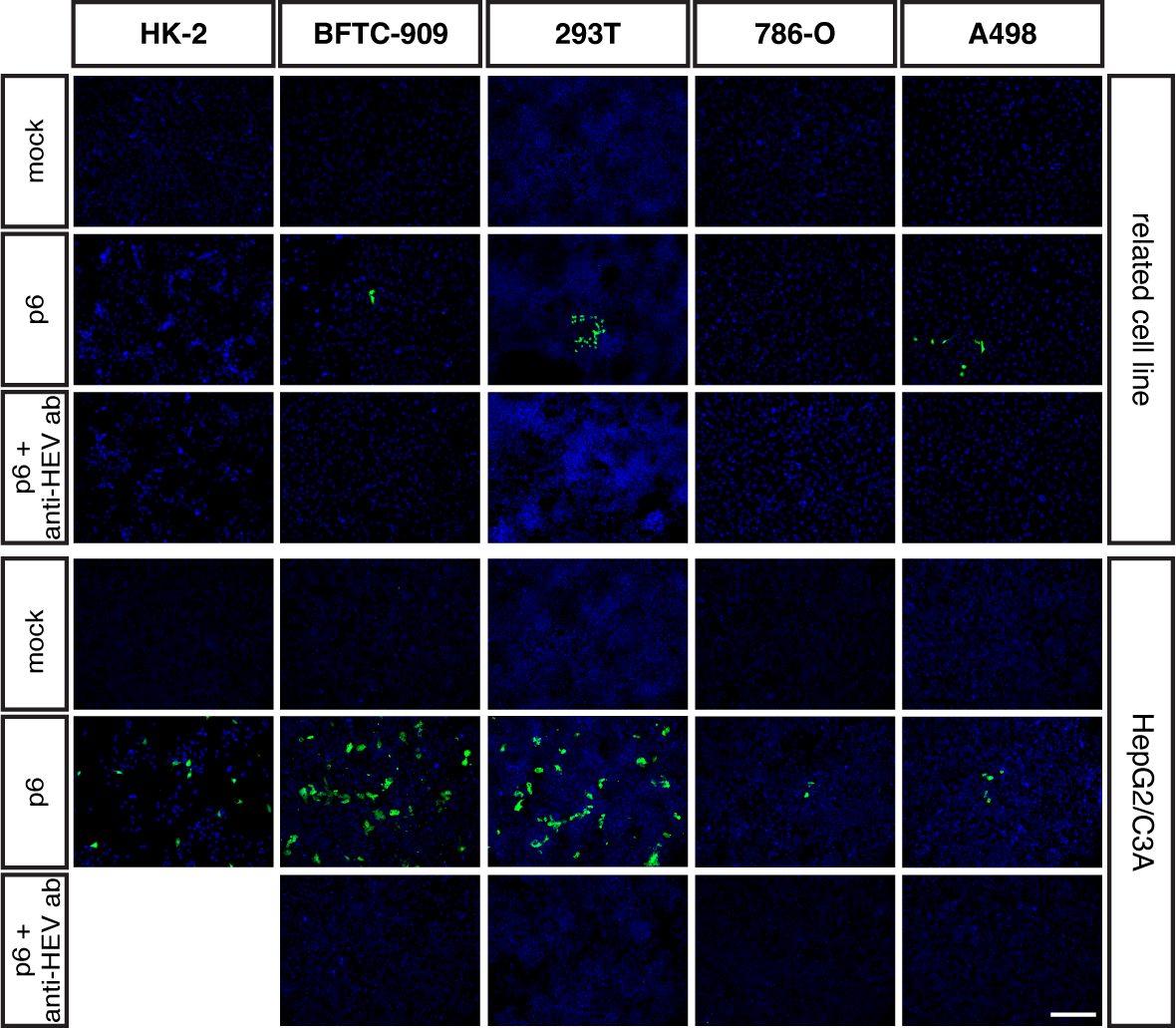


**Figure S4: Recapitulating the full HEV replicating cycle in kidney cells.** Virus produced in kidney cell lines was used for infection of HepG2/C3A cells and the same cell lines with neHEV. Five days after infection, the cells were immunostained for the ORF2-encoded capsid protein using an anti-capsid protein antibody. As negative control a neutralizing anti-HEV antibody at a concentration of 10 µg/mL was used. Scale bar denotes 200 µm.


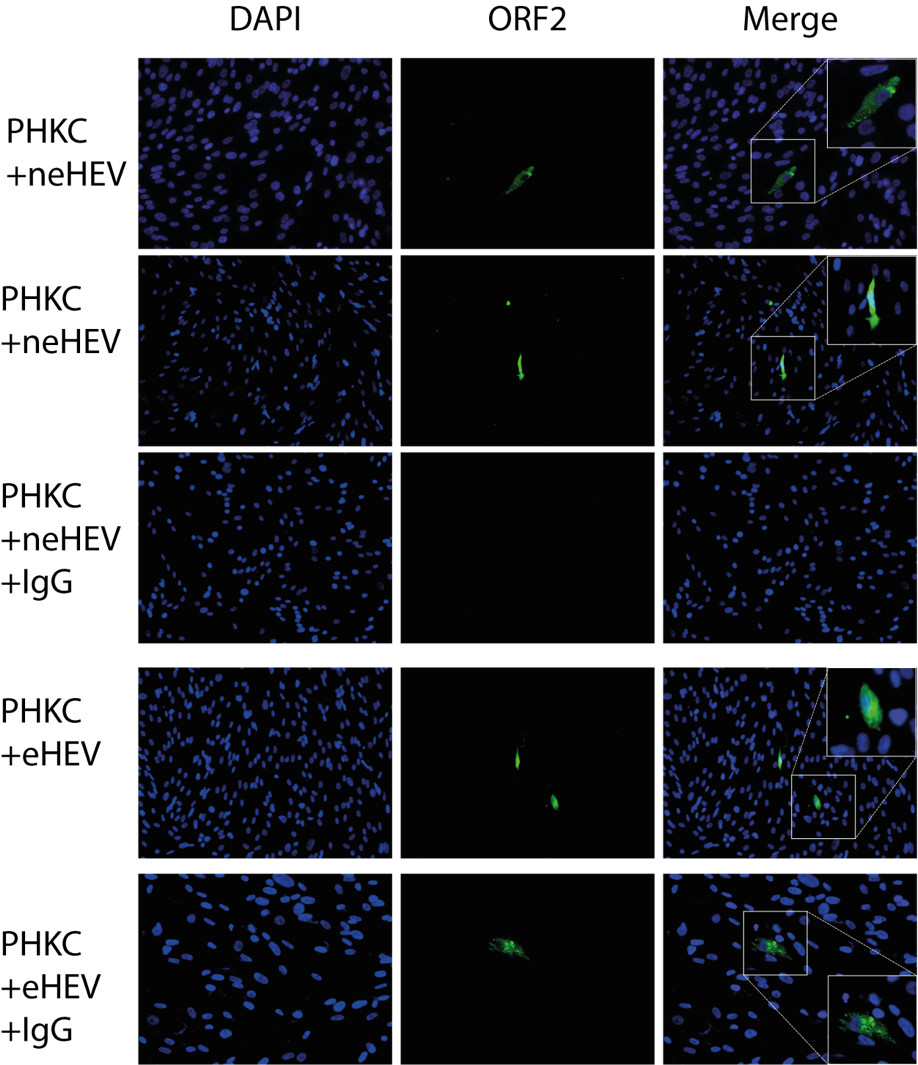


**Figure S5: Susceptibility of primary human and porcine kidney cells to HEV infection.** Primary porcine cells (PHKC) were inoculated with HEV and infectivity was determined by immunofluorescence staining of the capsid protein. Infection was done with non-enveloped HEV or enveloped HEV. Specificity was controlled by applying 1:100 IgG positive serum. Inlets show infected cells.


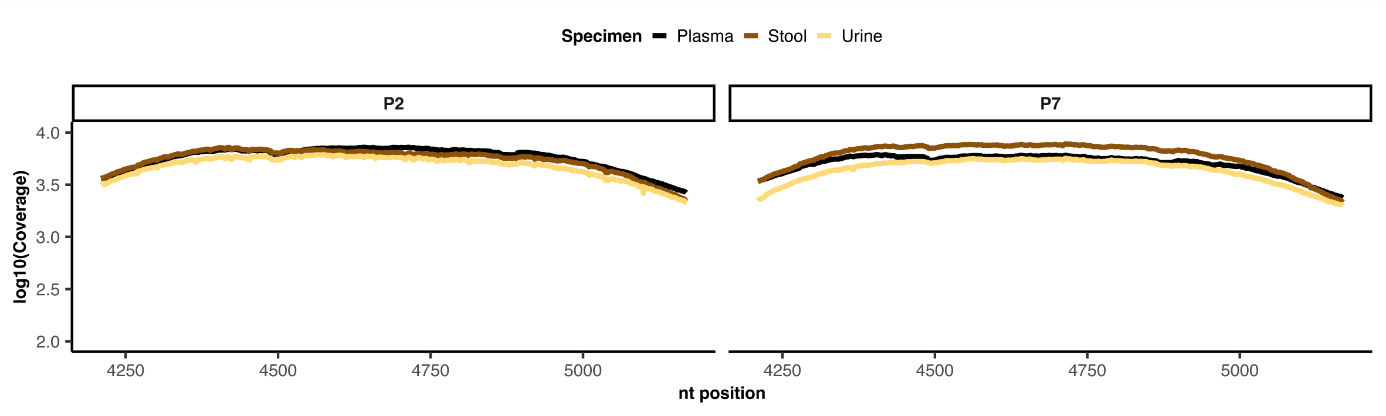


**Figure S6: Sequence coverage of the high-throughput sequencing data for the three specimens.** Coverage was determined for amplicons of patient 2 (P2) and patient 7 (P7) within the RNA-directed RNA polymerase coding region on nucleotide (nt) sequences.


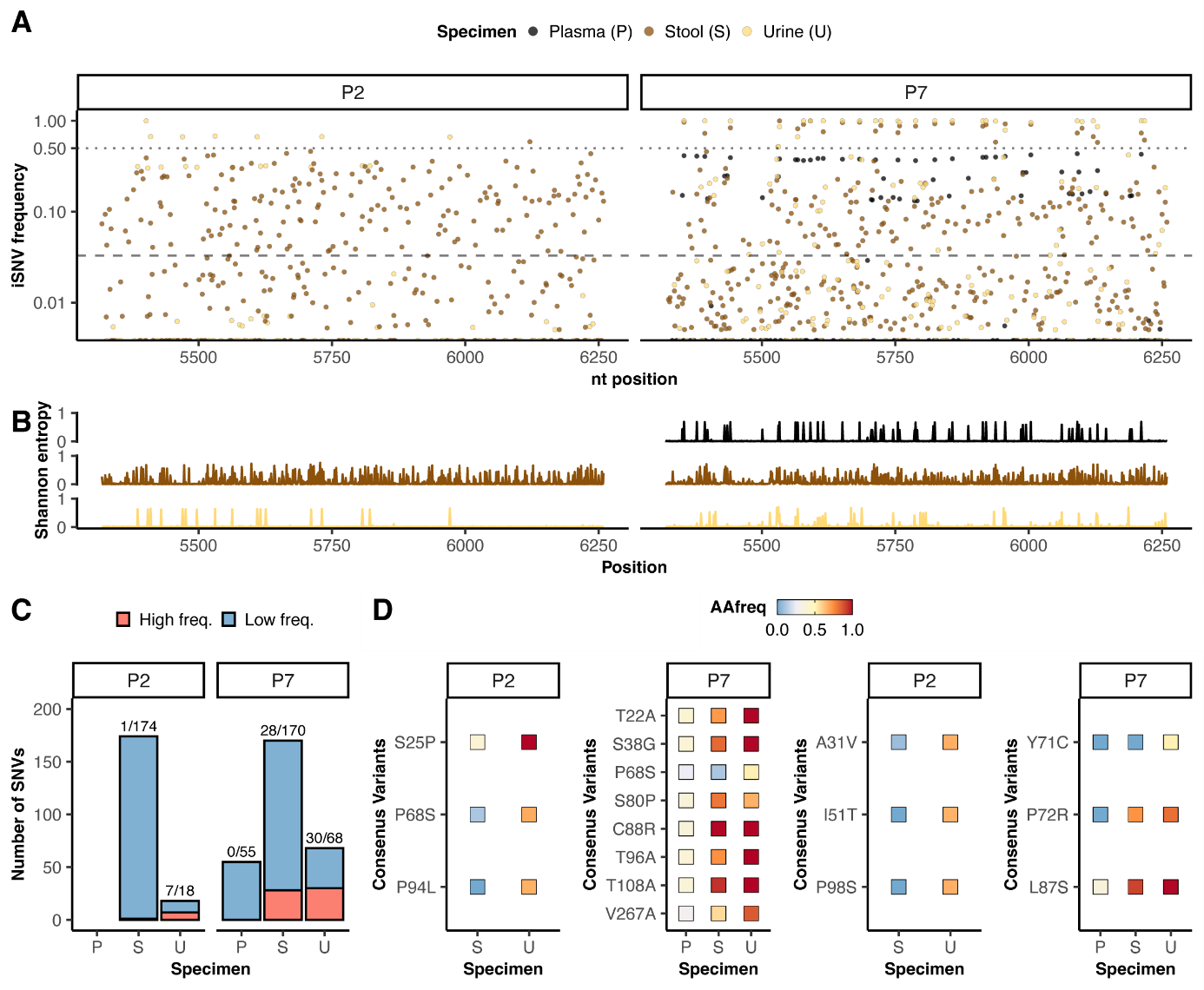


**Figure S7: Virus RNA sequencing from ORF2 and ORF3 encoding regions.** HEV ORF2 and ORF3 show mutational signatures of compartmentalization from different sources. (A) Intra-host single nucleotide frequencies (iSNV) of the N-terminal part of the ORF2 and ORF3 coding region of virus populations sampled from plasma (P, black), stool (S, brown) and urine (U, yellow) of patient 2 (P2) and patient 7 (P7). (B) Nucleotide sequence divergence summarised as Shannon entropy. (C) Quantification of low frequency (above 3.28%, blue) and high frequency (above 50%, red). (D) Frequency of amino acid (AA) substitutions within the ORF2 region (two left panels) and ORF3 (two right panels) of HEV strains in the three samples of both patients.

Table S1: Cell media composition.

| **Cell line** | **Medium** | **Supplements** |
| --- | --- | --- |
| HepG2 | DMEM complete  (Gibco) | Fetal calf serum (8%, Capricorn) Penicillin/Streptomycin (1%, Gibco)  L-Glutamine (2 mM, Gibco)  MEM NEAA (100x, Gibco) |
| BFTC-909 |  |  |
| 293T |  |  |
| 786-0 |  |  |
| HepG2/C3A | MEM ultra-low IgG FCS  (Gibco) | Ultra-low IgG fetal calf serum (10%, Gibco)  L-Glutamine (2 mM, Gibco)  MEM NEAA (100x, Gibco)  Sodium-pyruvate (1 mM, Gibco)  Gentamicin-sulfate (100 µg/ml, Gibco) |
| A498 | MEM complete  (Gibco) | Fetal calf serum (8%, Capricorn)  Penicillin/Streptomycin (1%, Gibco)  L-Glutamine (2 mM, Gibco)  MEM NEAA (100x, Gibco) |
| HK-2 | Keratinocyte Serum Free Medium  (Gibco) | KSF Supplement Kit (Gibco):  EGF Human Recombinant Bovine Pituitary Extract |
| Primary Human Renal Mixed Epithelial Cells | Renal Epithelial Cell Basal Medium  (ATCC) | Renal Epithelial Cell Growth Kit (ATCC):  Fetal bovine serum (0.5%)  Triiodthyronine (10 nM)  rh EGF (10 ng/ml)  Hydrocortisone Hemisuccinate (100 ng/ml)  rh Insulin (0.5 mg/ml)  Epinephrine (1 mM)  Transferrin (5 mg/ml)  L-Alanyl-L-Glutamine (2.4 mM)  Not in growth kit: Penicillin/Streptomycin (10 µg/ml, Gibco) Amphotericin B (25 ng/ml, Fungizone) |
| Primary Porcine Kidney Cells | DMEM-F12 (1:1) w/ GlutaMAX-I  (gibco) DMEM D-Valine for renal cell enrichment  (HIMEDIA) | Fetal calf serum (10%, gibco)  Penicillin/Streptomycin (50 U/ml / 50 µg/ml, 0.5%, gibco)  Insulin-Transferrin-Selenium (5 µg/ml, gibco) Amphotericin B (2.5 µg/ml, Fungizone) |
| Primary Porcine Hepatocytes | Williams’ Medium E w/ 2.2 g/L NaHCO_3,_ w/o L-Glutamine  (Biochrom) | Fetal calf serum (10%, gibco)  Penicillin/Streptomycin/Glutamine (1%, gibco)  7 µl Glucagon  175 µl ß-Mercaptoethanol (1:100)  12 ml of 15 ml stock solution  5 ml sodium-pyruvate (Gibco)  7.5 ml Hepes buffer  40 µl Soludecortin  400 µl human insulin (40 IE/ml) |

Table S2: Cell seeding densities.

| **Cell line** | **Density (cell/well)** | Plate Type | **Coating** | **Notes** |
| --- | --- | --- | --- | --- |
| HepG2/C3a | 1.5 - 2 x 10⁴ | 96-well | Collagen |  |
| A498 | 0.25 x 10⁴ | 96-well | Collagen |  |
| 786-O | 0.25 x 10⁴ | 96-well | Collagen |  |
| BFTC-909 | 0.25 x 10⁴ | 96-well | Collagen |  |
| HK-2 | 1.5 - 2 x 10⁴ | 96-well | Collagen |  |
| 293T | 3.25 x 10⁴ | 24-well | Poly-L-Lysin | Coverslip |
